# Supplementary material for: Tau filaments from multiple cases of sporadic and inherited Alzheimer’s disease adopt a common fold
Source: Acta Neuropathol. 2018 Oct 1;136(5):699–708. doi: 10.1007/s00401-018-1914-z (PMC6208733; doi:10.1007/s00401-018-1914-z)
Supplement: Supplementary file 1 — Online Resource 1 PHFs and SFs from the frontal cortex of AD case 2. (a,b) Fourier shell correlation curves between two independently refined half-maps (black line) and between the cryo-EM reconstruction and refined atomic model (red line) for PHFs (a) and SFs (b). (c,d) Local resolution estimates for PHF (c) and SF (d) reconstructions. (e,f) Views normal to the helical axis of PHF (e) and SF (f) reconstructions (PDF 2925 kb) [file 401_2018_1914_MOESM1_ESM.pdf]

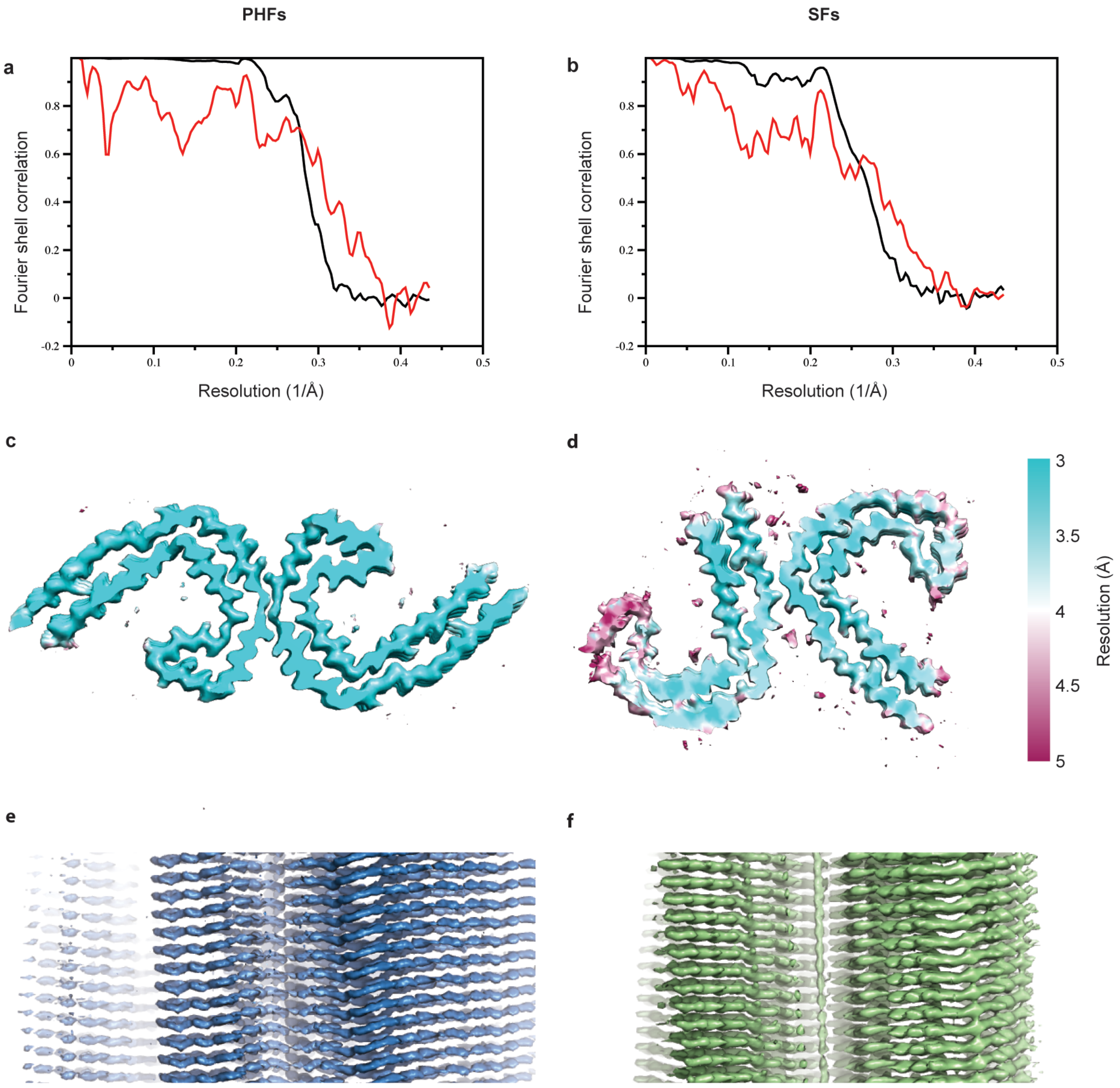

Online Resource 1 PHFs and SFs from the frontal cortex of AD case 2. (a,b) Fourier shell correlation curves between two independently refined half-maps (black line) and between the cryo-EM reconstruction and refined atomic model (red line) for PHFs (a) and SFs (b). (c,d) Local resolution estimates for PHF (c) and SF (d) reconstructions. (e,f) Views normal to the helical axis of PHF (e) and SF (f) reconstructions.
