# Supplementary material for: Tau filaments from multiple cases of sporadic and inherited Alzheimer’s disease adopt a common fold
Source: Acta Neuropathol. 2018 Oct 1;136(5):699–708. doi: 10.1007/s00401-018-1914-z (PMC6208733; doi:10.1007/s00401-018-1914-z)
Supplement: Supplementary file 2 — Online Resource 2 Electron cryo-microscopy structure determination (PDF 71 kb) [file 401_2018_1914_MOESM2_ESM.pdf]

Online Resource 2: Electron cryo-microscopy structure determination

	Case 2		Case 3		Case 16	
**Data Collection**	**PHFs**	**SFs**	**PHFs**	**SFs**	**PHFs**	**SFs**
Magnification	×105,000	×105,000	×105,000	×105,000	×165,000	×165,000
Defocus range (μm)	-1.7 to -2.8	-1.7 to -2.8	-1.7 to -2.8	-1.7 to -2.8	-1.7 to -2.8	-1.7 to -2.8
Voltage (kV)	300	300	300	300	300	300
Microscope	Titan Krios	Titan Krios	Titan Krios	Titan Krios	Titan Krios	Titan Krios
Detector	K2 Summit	K2 Summit	K2 Summit	K2 Summit	K2 Summit	K2 Summit
Frame exposure time (ms)	200	200	200	200	250	250
Number of frames	50	50	50	50	44	44
Total dose (e^-/Å^{-2})	60	60	60	60	57.5	57.5
Pixel size (Å)	1.15	1.15	1.15	1.15	0.85*	0.85*
**Reconstruction**						
Box size (pixel)	270	270	270	270	200	200
Inter-box distance (Å)	28	28	28	28	20	20
Segments extracted	197,500	62,782	35,400	35,400	190,409	190,409
Segments after Class2D	95,917	29,536	22,150	12,690	115,669	47,437
Segments after Class3D	n/a	27,103	n/a	n/a	54,900	29,594
Resolution (Å)	3.2	3.3	4.7	8.4	3.8	4.2
B-factor (Å^2)	-90	-61	-152	n/a	-87	-58
Helical rise (Å)	2.37	4.76	2.36	4.74	2.39	4.84
Helical twist (°)	179.45	-1.04	179.40	-1.05	179.45	-1.02

* For image processing, the segments for case 16 were downscaled to a pixel size of 1.53 Å.
