# Supplementary material for: Tau filaments from multiple cases of sporadic and inherited Alzheimer’s disease adopt a common fold
Source: Acta Neuropathol. 2018 Oct 1;136(5):699–708. doi: 10.1007/s00401-018-1914-z (PMC6208733; doi:10.1007/s00401-018-1914-z)
Supplement: Supplementary file 3 — Online Resource 3 Patient 2 PHF and SF model statistics (PDF 57 kb) [file 401_2018_1914_MOESM3_ESM.pdf]

Online Resource 3: Patient 2 PHF and SF model statistics

Refinement	PHFs	SFs
Initial model used (PDB code)	5o3l	5o3t
Model resolution (Å)	3.2	3.3
FSC threshold	0.5	0.5
Map sharpening *B* factor ($Å^2$)	-90	-61
Model composition		
Non-hydrogen atoms	587	587
Protein residues	456	456
Average *B* factor ($Å^2$)	72.8	82.0
R.m.s. deviations		
Bond lengths (Å)	0.019	0.016
Bond angles (°)	1.37	1.11
Validation		
MolProbity score	1.90	2.00
Clashscore	5.59	6.71
Poor rotamers (%)	0	0
Ramachandran plot		
Favored (%)	88	87
Allowed (%)	100	100
Disallowed (%)	0	0
