# Supplementary material for: Tau filaments from multiple cases of sporadic and inherited Alzheimer’s disease adopt a common fold
Source: Acta Neuropathol. 2018 Oct 1;136(5):699–708. doi: 10.1007/s00401-018-1914-z (PMC6208733; doi:10.1007/s00401-018-1914-z)
Supplement: Supplementary file 4 — Online Resource 4 Protofilament structures of AD cases 1 and 2. (a,b) Overlay of backbone atoms of the protofilament structures of PHFs (a) and SFs (b) from AD cases 1 (blue) and 2 (green). (c,d) As in (a,b), but showing all atoms (PDF 1316 kb) [file 401_2018_1914_MOESM4_ESM.pdf]

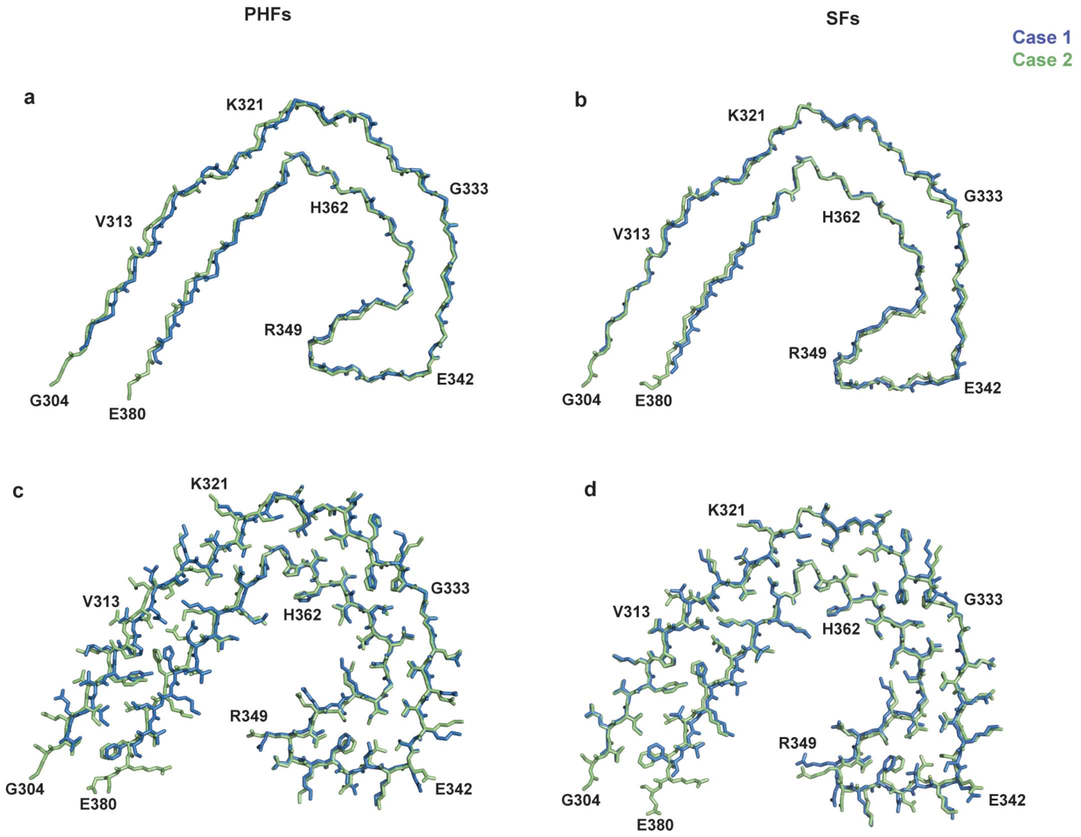

Online Resource 4 Protofilament structures of AD cases 1 and 2. (a,b) Overlay of backbone atoms of the protofilament structures of PHFs (a) and SFs (b) from AD cases 1 (blue) and 2 (green). (c,d) As in (a,b), but showing all atoms.
