# Supplementary material for: Tau filaments from multiple cases of sporadic and inherited Alzheimer’s disease adopt a common fold
Source: Acta Neuropathol. 2018 Oct 1;136(5):699–708. doi: 10.1007/s00401-018-1914-z (PMC6208733; doi:10.1007/s00401-018-1914-z)
Supplement: Supplementary file 5 — Online Resource 5 Protofilament interface in PHFs. Packing between residues 331KPGGGQV337 of the two protofilaments. Inter-protofilament hydrogen bonds are present between G333 and G334, and between Q336 and the backbone carbonyl of K331. Additional densities between the side chains of K331 and the backbone atoms of V337 are highlighted with dashed orange outlines (PDF 1195 kb) [file 401_2018_1914_MOESM5_ESM.pdf]

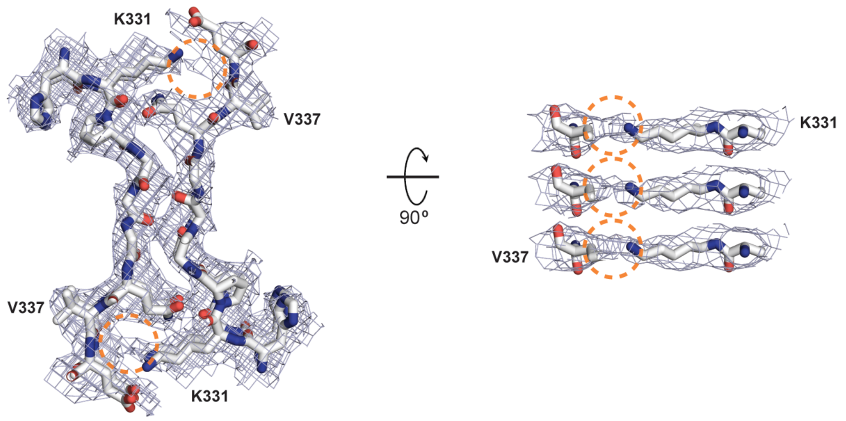

Online Resource 5 Protofilament interface in PHFs. Packing between residues ^{331}KPGGGQV^{337} of the two protofilaments. Inter-protofilament hydrogen bonds are present between G333 and G334, and between Q336 and the backbone carbonyl of K331. Additional densities between the side chains of K331 and the backbone atoms of V337 are highlighted with dashed orange outlines.
