# Supplementary material for: Tau filaments from multiple cases of sporadic and inherited Alzheimer’s disease adopt a common fold
Source: Acta Neuropathol. 2018 Oct 1;136(5):699–708. doi: 10.1007/s00401-018-1914-z (PMC6208733; doi:10.1007/s00401-018-1914-z)
Supplement: Supplementary file 6 — Online Resource 6 Western blot with BR136. Western blot with antibody BR136 (against residues 244–257 from R1 of tau) of the sarkosyl-insoluble fraction from the frontal cortex of AD case 1 (PDF 724 kb) [file 401_2018_1914_MOESM6_ESM.pdf]

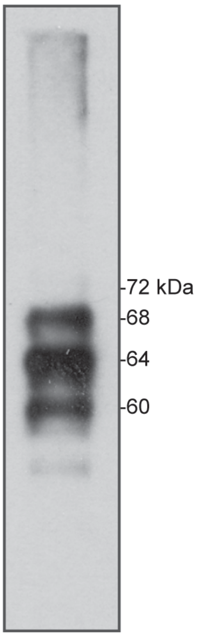

Online Resource 6 Western blot with BR136. Western blot with antibody BR136 (against residues 244–257 from R1 of tau) of the sarkosyl-insoluble fraction from the frontal cortex of AD case 1.
