# Supplementary material for: Tau filaments from multiple cases of sporadic and inherited Alzheimer’s disease adopt a common fold
Source: Acta Neuropathol. 2018 Oct 1;136(5):699–708. doi: 10.1007/s00401-018-1914-z (PMC6208733; doi:10.1007/s00401-018-1914-z)
Supplement: Supplementary file 7 — Online Resource 7 Immuno-EM labelling of PHFs and SFs (PDF 72 kb) [file 401_2018_1914_MOESM7_ESM.pdf]

Online Resource 7: Immuno-EM labelling of PHFs and SFs

		R1		R2		R3		R4	
**Case**	**Brain region**	-	+	-	+	-	+	-	+
1	Frontal cortex	Yes	No	Yes	No	No	No	No	No
2	Frontal cortex	Yes	No	Yes	No	No	No	No	No
3	Frontal cortex	Yes	No	Yes	No	No	No	No	No
4	Frontal cortex	Yes	No	Yes	No	No	No	No	No
5	Frontal cortex	Yes	No	Yes	No	No	No	No	No
6	Frontal cortex	Yes	No	Yes	No	No	No	No	No
7	Frontal cortex	Yes	No	Yes	No	No	No	No	No
8	Frontal cortex	Yes	No	Yes	No	No	No	No	No
9	Frontal cortex	Yes	No	Yes	No	No	No	No	No
10	Frontal cortex	Yes	No	Yes	No	No	No	No	No
11	Frontal cortex	Yes	No	Yes	No	No	No	No	No
12	Frontal cortex	Yes	No	Yes	No	No	No	No	No
13	Frontal cortex	Yes	No	Yes	No	No	No	No	No
14	Frontal cortex	Yes	No	Yes	No	No	No	No	No
15	Frontal cortex	Yes	No	Yes	No	No	No	No	No
1	Temporal cortex	Yes	No	Yes	No	No	No	No	No
1	Occipital cortex	Yes	No	Yes	No	No	No	No	No
1	Parietal cortex	Yes	No	Yes	No	No	No	No	No
1	Cingulate cortex	Yes	No	Yes	No	No	No	No	No
1	Thalamus	Yes	No	Yes	No	No	No	No	No
1	S. innominata	Yes	No	Yes	No	No	No	No	No
1	Putamen	Yes	No	Yes	No	No	No	No	No
16	Frontal cortex	Yes	No	Yes	No	No	No	No	No
17	Frontal cortex	Yes	No	Yes	No	No	No	No	No
18	Frontal cortex	Yes	No	Yes	No	No	No	No	No
18	Occipital cortex	Yes	No	Yes	No	No	No	No	No
19	Frontal cortex	Yes	No	Yes	No	No	No	No	No
19	Occipital cortex	Yes	No	Yes	No	No	No	No	No

-, no pronase; +, pronase. Yes, antibody labelling; No, no antibody labelling. Cases 1, 2, 3 and 16 were used for cryo-EM (highlighted in yellow).
